# Quality of life questionnaires validate a remote approach to ophthalmic management of primary Sjögren’s syndrome

**DOI:** 10.1038/s41598-022-23676-x

**Published:** 2022-11-05

**Authors:** E. Greenan, Q. Pilson, J. Ní Gabhann-Dromgoole, C. C. Murphy

**Affiliations:** 1 Royal Victoria Eye and Ear Hospital, Adelaide Rd, Dublin 2, D02 XK51 Ireland; 2 Department of Ophthalmology, RCSI, University of Medicine and Health Sciences, 123 St Stephen’s Green, Dublin 2, D02 YN77 Ireland; 3 School of Pharmacy and Biomolecular Sciences, RCSI, University of Medicine and Health Sciences, 123 St Stephen’s Green, Dublin 2, D02 YN77 Ireland; 4 Department of Ophthalmology, Stepping Hill Hospital, Poplar Grove, Stockport, Cheshire, SK2 7JE England

**Keywords:** Corneal diseases, Eye manifestations, Autoimmunity, Rheumatology, Quality of life

## Abstract

Those with underlying autoimmune conditions were met with unparalleled challenges and were disproportionately affected by the COVID-19 pandemic. As such, we aimed to measure the impact of the pandemic on symptoms and the health and vision related quality of life (HR-QoL, VR-QoL) in patients with Primary Sjögren's Syndrome (pSS). Nineteen (55.9%) participants returned questionnaires for analysis, (17 female and 2 male, 61.6 years ± 9.9). There was no significant change in participants HR-QoL or VR-QoL, indicating that those with pSS remained resilient with regard to their physical and mental health throughout the pandemic. Furthermore, QoL was maintained despite 73.7% of participants having had outpatient appointments cancelled, delayed or rescheduled. Participants reported a lower QoL and feeling tenser in the COV19-QoL (3.3 ± 1.4 and 3.2 ± 1.3) representing feelings of apprehension and stress felt amongst the general population since the pandemic. Overall, and in spite of the concern caused by the COVID-19 pandemic for patients with autoimmune diseases, the health and well-being of patients with pSS remained stable. These findings strongly support the use of validated HR and VR-QoL questionnaires as an adjunct to the telemedicine consultation when assessing patients with pSS, offering an alternative to face-to-face consultations in post-pandemic era.

## Introduction

Primary Sjögren’s Syndrome (pSS) is a complex autoimmune disease that primary affects the lacrimal and salivary glands leading to a sicca complex often accompanied by joint pain and fatigue^1^. The condition predominately affects women in a 9:1 ratio compared with men, with symptoms manifesting most commonly from the ages of 53–60 years of age^2,3^. Although the clinical presentation of pSS is most typically that of xerostomia and keratoconjunctivitis sicca nearly half will go on to develop extra-glandular disease resulting from immune complex deposition in tissues^4^. Due to the complexity of the disease, chronicity of symptoms and systemic involvement, patients with pSS will often require care from different specialists, requiring a higher median of outpatient visits per year than the general population^5^.

The first case of the novel Severe Acute Respiratory Syndrome coronavirus (SARS-CoV)-2 in Ireland was reported on the 29th of February 2020^6^. At the time of writing, the coronavirus disease 2019 (COVID-19) pandemic has so far led to over 233 million cases and more than 4.75 million deaths^7^. Those infected can be asymptomatic, while others can develop severe pneumonia and life-threatening respiratory failure^8,9^. Due to the underlying immune dysfunction and frequent use of immunosuppressive medications, patients with autoimmune conditions like pSS are considered at an increased risk of poor outcomes from COVID-19 infection^10^.

The public health response to the COVID-19 focused on containment, with the implementation of national and regional lockdowns, social distancing and the closure of schools, work places and public areas^11^. Under unprecedented conditions, the healthcare system was restructured to prioritise hospital capacity for critical COVID-19 patients. Elective care and outpatient services were cancelled, rescheduled or delayed, with a rapid re-organisation of chronic disease management^12^. Furthermore, for many services telemedicine became the predominant method for the medical assessment and the delivery of care^13^. This presented a balance of protecting patients with chronic diseases from the risk of infection with COVID-19 while also providing care, preventing disease progression, and managing their disease and symptoms effectively.

Thus, we sought to measure the impact of the SARS-CoV-2 pandemic on the perceived health and vision related quality of life (HR-QoL and VR-QoL) in a cohort of patients with pSS.

## Results

A total of nineteen participants (55.9%) returned questionnaires for analysis (17 female and 2 male) with a mean age of 61.6 years (± 9.9 years). Of the responders, seven were taking immunosuppressant agents (36.8%). The mean duration of disease symptoms was 3.7 years (± 14.3, range 0.2–28.0 years) and the mean EULAR Sjögren's syndrome disease activity index (ESSDAI) score of respondents was 6.0 (± 8.3, range 0–14). There was no statistically significant difference between those who returned questionnaires and those who did not in terms of characteristics, disease duration and ESSDAI scores as shown in Table 1.

**Table 1:** Comparing the demographic and disease characteristics of those that responded and those that did not return questionnaires.

	Responder	Non responder	P value
Ethnicity			
White Caucasian	19 (100%)	15 (100%)	
Gender			
Male	17	11	> 0.9999
Female	2	4	
Age (yrs)	61.9 ± 9.9	60.9 ± 12.1	> 0.9999
Disease duration (yrs)	3.7 ± 14.3	5.6 ± 5.1	0.59
Immunosuppression	7 (36.8%)	7 (46.7%)	> 0.9999
ESSDAI	6 ± 8.3	8.6 ± 7.6	> 0.9999

ESSDAI, EULAR Sjögren's syndrome disease activity index; yrs, years.

### Ocular Surface Disease Index Questionnaires (OSDI)

**Figure 1:**
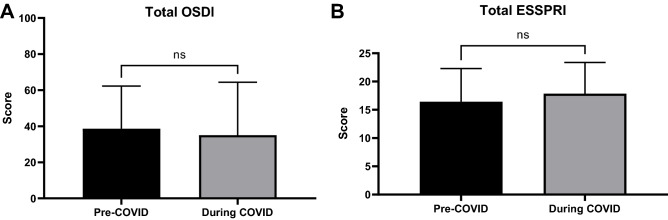
Comparison of pre COVID-19 and during COVID-19 overall (**A**) OSDI scores and (**B**) overall ESSPRI scores. OSDI, Ocular Surface Disease Index; ESSPRI, EULAR Sjögren’s Syndrome Patient Reported Index.

Before the COVID-19 pandemic, 15.8% (n = 3) of participants had normal OSDI overall scores, 15.8% (n = 3) had scores within the mild range, 10.5% (n = 2) had moderate scores and 57.9% (n = 11) had severe scores. Eighteen months after the pandemic began OSDI scores shifted left with 26.3% (n = 5) recording normal overall scores, 10.5% (n = 2) mild ranging scores, 26.3% (n = 5) within the moderate and 36.8% (n = 7) severe range scores. None of the changes in OSDI score were statistically significant. A breakdown of these results is represented in Table 2, and visually illustrated in Fig. 1A.

Additional/bespoke survey questions found that during the pandemic 42.1% (n = 8) of respondents had ophthalmology outpatient appointments either cancelled, delayed or rescheduled. There was no significant difference in scores between those whose appointments were and were not affected (mean 33.5 ± 27.7 vs mean 33.8 ± 31.6, p = 0.44). When comparing pre and during COVID-19 questionnaires, there was no significant change in the OSDI overall score for those who had their appointments affected (mean 41.0 ± 16.3 vs mean 37.52 ± 27.9, p = 0.64) and those who did not (mean 36.9 ± 28.6 vs mean 33.8 ± 31.6, p = 0.48).

### EULAR Sjögren’s syndrome patient reported index (ESSPRI)

In relation to the ESSPRI, 63.2% (n = 12) of participants had a worsening of their total score (4.4 ± 2.9, range 1–9), while no change or a decrease in the score was experienced by 36.8% (n = 7) of participants (2.1 ± 2.9, range 0–9). Overall, there was no significant change in ESSPRI subscale or total scores comparing pre- and during-pandemic results. This is illustrated in Fig. 1B and expanded upon in Table 2.

**Table 2:** Comparing pre COVID-19 and during COVID-19 results of the OSDI and ESSPRI subscales and overall scores (Wilcoxon test).

Questionnaire subsection	Pre COVID-19 (mean ± SD)	During COVID-19 (mean ± SD)	P value
OSDI symptoms	39.3 ± 21.5	38.9 ± 28.3	0.72
OSDI function	26.3 ± 27.4	23.79 ± 30.0	0.57
OSDI environment	53.5 ± 39.1	43.9 ± 46.1	0.41
OSDI total	38.6 ± 28.3	35.4 ± 29.3	0.12
ESSPRI dryness	6.3 ± 2.1	6.7 ± 1.9	0.88
ESSPRI fatigue	5.8 ± 2.9	6.0 ± 2.4	> 0.99
ESSPRI pain	4.3 ± 3.1	5.1 ± 3.2	0.73
ESSPRI total	16.4 ± 5.9	17.8 ± 5.5	0.64

ESSPRI, EULAR Sjögren’s Syndrome Patient Reported Index; OSDI, Ocular Surface Disease Index.

Ten participants (52.6%) had had their specialist appointments with rheumatology, ophthalmology or oral medicine affected through cancellation, delay or rescheduling during the pandemic lockdowns. There was no statistically significant change in the pre and during COVID-19 ESSPRI questionnaire total scores between those who had outpatient appointments affected (mean 19.7 ± 4.9 vs mean 18.8 ± 5.2) and those who did not (mean 12.8 ± 4.8 vs 16.8 ± 5.3).

### National Eye Institute Visual Function-25 Questionnaire (NEI VF-25)

**Figure 2:**
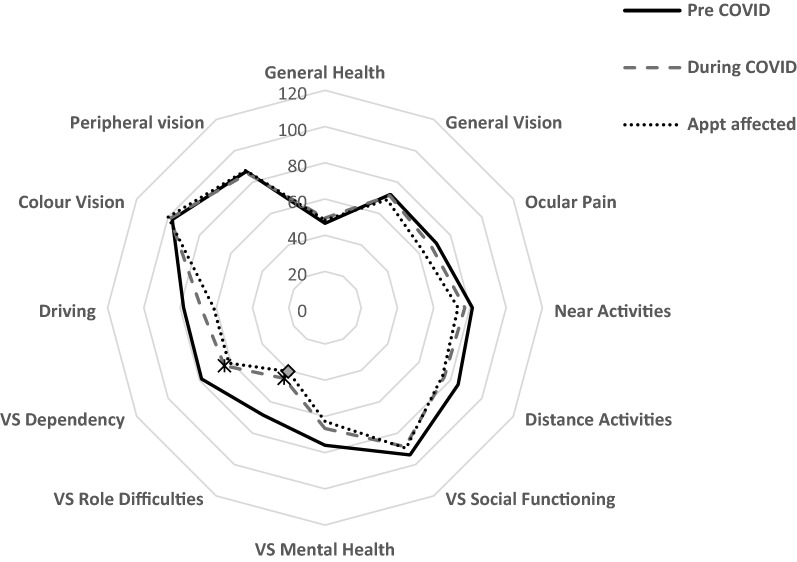
Radar chart comparing the NEI VF-25 results of pre COVID-19 and during COVID-19 NEI VF-25 results (Wilcoxon test) and participants whose outpatient appointments with ophthalmology were affected by the COVID-19 related lockdown (Mann–Whitney test). VS, vision specific, *p < 0.01, ◊p < 0.03.

When comparing pre and during COVID-19 NEI VF-25 questionnaire results, participants responses were significantly lower only in relation to vision specific dependency and vision specific role difficulties (68.4 ± 24.4 vs 45.1 ± 8.2, p < 0.01 and 78.7 ± 23.4 vs 64.01 ± 15.5, p < 0.01). Of those who had participated in the study, 42.1% (n = 8) had ophthalmology outpatient appointments either cancelled, delayed or rescheduled. With the exception of vision specific role difficulties (40.6 ± 10.2 vs 48.3 ± 4.9, p = 0.03), there was no significant difference in the NEI VF-25 subscale results between those whose appointments were affected and those that were not. This is visually represented in the radar chart in Fig. 2.

### Short form 36 (SF-36)

When weighted and compared to normative data, participants with pSS had a significantly lower perceived HR-QoL in all scales of the SF-36, both pre COVID-19 (MD = 20.0 ± 8.6; t (7) = 6.6, p < 0.001) and during COVID-19 total (MD = 20.5 ± 10.6; t (7) = 5.5, p = 0.001). There was no significant difference between participants’ subscale scores comparing pre COVID-19 and during COVID-19 values (MD = 0.5 ± 4.0; t (7) = 0.37, p = 0.72). Eleven participants (57.9%) had appointments with their dentist, general practitioner, ophthalmologist or rheumatologist affected by the pandemic. A comparison of those whose appointments were and were not affected by the pandemic did not show a significant difference in any of the subsections of the SF-36 questionnaire (MD = 3.47 ± 11.5; t (7) = 0.86, p = 0.42). This is demonstrated in Table 3.

**Table 3:** SF-36 subscale results (mean ± SD) and component summaries from normative data and from those with pSS, including pre-COVID-19, during COVID-19, and the results of those whose appointments had been cancelled, delayed or reschedule during the pandemic.

SF 36 subscale	Normative data mean (SD)	Pre COVID-19 pSS mean (SD)	During COVID-19 pSS mean (SD)	Appts affected mean (SD)
PF	82.2 (22.9)	57.1 (25.0)	52.9 (26.3)	55.9 (25.0)
RP	80.5 (34.5)	50 (43.3)	44.7 (40.5)	28.6 (37.7)
BP	77.6 (26.4)	63.2 (30.0)	60.5 (25.2)	52.3 (16.9)
GH	73.8 (21.1)	41.6 (29.0)	43.7 (23.0)	37.7 (16.9)
VT	64.8 (20.4)	43.4 (20.8)	41.6 (20.3)	41.8 (18.1)
SF	84.1 (23.1)	69.7 (29.3)	77.0 (19.2)	70.5 (21.1)
RE	83.2 (31.8)	71.9 (43.4)	71.9 (40.5)	66.7 (42.2)
MH	77.8 (16.5)	67.2 (18.5)	67.6 (17.9)	65.1 (18.1)
PCS	49.4	37.3	35.8	36.8
MCS	50.4	46.4	47.9	47.3

PF, physical function; RP, role limitation due to physical health; BP, bodily pain; GH, general health; VT, vitality; SF, social function; RE, role limitation due to emotional health; MH, mental health; PCS, physical component summary; MCS, mental component summary.

### COV19-QoL Questionnaire

Table 4 outlines the six COV19-QoL questions that were posed to participants to assess the perceived personal impact of the pandemic. Participants scored highest on items relating to their overall sense of QoL (3.3 ± 1.4), feeling more tense than before (3.2 ± 1.3) and perception of physical health deterioration (3.1 ± 1.2). The lowest perceived impact was found relating to personal safety (2.3 ± 1.3).

**Table 4:** Personal experience of participants with Primary Sjögren’s Syndrome during the COVID-19 pandemic.

	Mean	SD
I think my quality of life is lower than before	3.3	1.4
I think my mental health has deteriorated	2.8	1.2
I think my physical health may have deteriorated	3.1	1.2
I feel more tense than before	3.2	1.3
I feel more depressed than before	2.5	1.1
I feel that my personal safety is at risk	2.3	1.3
Total	2.9	1.0

The association of participants personal experience during the pandemic to that of symptom severity and both HR-QoL and VR-QoL was examined through correlation analysis. The COV19-QoL correlated with post NEI VF-25 general health (r(17) = − 0.46, p < 0.05), and the SF-36 subsections of physical function (r(19) =  − 0.77, p < 0.001), vitality (r(17) =  − 0.71, p < 0.001), and mental health (r(17) =  − 0.60, p = 0.006). However, there was no significant association between the COV19-QoL total scores with that of the changes between pre and during COVID-19 results from OSDI, ESSPRI, NEI VF-25 and SF-36 questionnaire items.

Analysis of the additional questions asked to participants showed that the majority of participants had no disruption to their prescribed medication regime (n = 18). Only 16.7% (n = 2) reducing or discontinuing their immunosuppression during the period in light of the pandemic. Just over half of the participants, 52.6% (n = 10), experienced a worsening of their sicca symptoms due to the wearing of masks.

## Discussion

The COVID-19 pandemic has led to unprecedented challenges to those vulnerable patients with underlying autoimmune disorders. However, our study has demonstrated that those with pSS have remained physically and mentally resilient throughout the course of the pandemic.

Through this investigation we have shown that there was no statically significant change in the SF-36 questionnaire results of participants from their baseline, suggesting that pSS patients were able to cope with the sustained adjustments and associated physical and mental strain of the pandemic. In contrast to the results from this measure of HR-QoL, participants reported experiencing both a lower quality of life, and feeling tenser than they did pre-pandemic in the COV19 QoL survey. This may reflect the overall toil and stress felt across the general population since the outbreak of the virus. Such feelings of uncertainty, anxiety and fear have been reported by authors amongst patients with pSS and rheumatic diseases^14,15^.

Like with many other chronic diseases, the COVID-19 pandemic has affected the management and treatment of patients with pSS, both with regard to provision of care and the ongoing management of the disease and symptoms. Due to lockdowns and fears of infection, healthcare providers and clinicians were forced to adopt and implement telemedicine services in a short space of time. In this study, over 73% of participants had outpatient appointments negatively impacted through cancellation, delay or rescheduling. However, despite this change in practice, participant’s perception of disease severity as well as HR-QoL and VR-QoL results did not change, with the exception of VS dependency and VS role difficulties. This implies that participants’ disease and sicca symptoms remained stable throughout the course of the pandemic. This is in contrast to the results from Carubbi et al. which showed that those with pSS in Italy suffered from a worsening of symptoms during the SARS-CoV2 outbreak^16^.

The use of validated questionnaires in previous studies has suggested that it is possible to evaluate and manage ocular surface disease in a virtual clinic setting^17,18^. The lessons learned and experience gained during the pandemic period could be used to integrate remote care into routine clinical practice. This could offer an alternative to face-to-face consultations to be used when disease activity of patients with pSS is low and stable. It would allow patients to avoid the unnecessary burden of travel and the social and work-related costs of attending outpatient appointments in person. To facilitate this, it is essential that screening and disease evaluation tools are validated to ensure that patients’ healthcare needs are adequately assessed for such a service to be utilised in the post pandemic era. The findings of the present study strongly support telemedicine delivered care to patients with mild to moderate pSS disease activity.

This study is limited by the low number of completed surveys by participants during COVID-19 pandemic (n = 19, 55.9% of total). However, by using four validated and widely used self-reported questionnaires and a bespoke COVID-19 survey, we believe that this study is a unique and important reflection of the impact that the pandemic has had on patients with pSS. Additionally, while this study places a strong emphasis on bioclinical parameters, the possible impact of demographic, physiological, psychological, social, and environmental factors on patient reported outcomes was not assessed^19^.

To conclude, the COVID-19 outbreak has resulted in unrivalled challenges for patients with pSS in relation to their well-being and access to healthcare. This study has shown that patients with pSS have remained resilient in relation to their physical and mental health throughout the course of the pandemic to date. Furthermore, the findings of this study strongly support the use of validated HR-QoL and VR-QoL questionnaires as an adjunct to the telemedicine consultation when assessing patients with pSS, offering an alternative to face-to-face consultations in a post pandemic era.

## Materials and methods

### Participants

Thirty-four participants with a confirmed diagnosis of pSS in accordance with the 2016 ACR EULAR diagnostic criteria were contacted in relation to the study^20^. Those contacted were pSS patients who attended a tertiary referral ophthalmic centre. Participants were from throughout the country and are considered to be representative of the national experience of those living with pSS. The recruitment of participants was limited to the data that was on record prior to February 2020 and the beginning of the pandemic.

All participants provided their informed consent and were sent HR-QoL and VR-QoL questionnaires as well as a COVID-19 specific questionnaire, COV19-QoL. Questionnaires were sent to those participants in June of 2021. By then, the country and its population had lived through 18 months of the pandemic and associated restrictions, and was beginning a phased reopening. HR-QoL and VR-QoL results were compared to the same patients’ results obtained in an earlier study prior to the pandemic. These pre-pandemic questionnaires were completed as part of a clinical study in 2014, which was then restarted in 2019. Patients were not participating in a clinical trial. Participants were sent reminder letters to return the questionnaires four weeks after having been initially contacted.

### Questionnaires

Five self-administered questionnaires were used, two to assess symptom severity, another two to measure VR-QoL and HR-QoL, and finally the COV19-QoL questionnaire.

#### Ocular surface disease index (OSDI)

The OSDI is a validated 12-item questionnaire that is used to effectively assess the severity of dry eye disease (DED)^21^. The self-assessment tool has three subsections; ocular symptoms, vision related function and environmental triggers. A final score out of 100 is calculated, with 0–12 representing normal, 13–22 as mild DED, 23–32 as moderate disease and greater than 33 representing severe DED.

#### EULAR Sjögren’s syndrome patient reported index (ESSPRI)

The ESSPRI is designed to measure the three main symptoms of pSS; dryness, fatigue, and pain. They are rated on a scale from 1 to 10, with a higher score indicating more severe symptoms^22^.

#### National Eye Institute Visual Function (NEI VFQ-25)

The NEI VFQ-25 is a general questionnaire used to measure the impact of chronic ocular diseases on VR-QoL^23^. The questionnaire measures general health and eleven visual function domains; general vision, ocular pain, near and distance vision, vision specific (VS) social functioning, mental health, role difficulties and dependency, driving, colour and peripheral vision. Each subscale is converted into a total score ranging from 0 to 100, with a higher score indicating better VR-QoL.

#### Short form-36 (SF-36)

The SF-36 is a generic measure of perceived HR-QoL in eight domains of patients day to day life. These include physical function, role physical, bodily pain, general health, vitality, social function, role emotional, and mental health. Subscales are calculated ranging from 0 to 100 within each subscale. Higher scores indicated better HR-QoL. Physical Component Summary (PCS) and Mental Component Summary (MCS) scores were calculated as recommended by Ware et al.^24^. Results were weighted against gender and age, and compared to normative data^25^.

#### COV19-QoL

The COV19-QoL assesses the respondent’s QoL in the past week during the COVID-19 pandemic^26^. It has 6 items rated on a 5-point Likert-type scale ranging from 1 (totally agree) to 5 (totally disagree). The items (i.e. statements) cover main areas of QoL regarding mental health. A higher score indicates that the perceived effect of the pandemic on a person’s quality of life is higher. Additional questions were asked to further measure the personal experience of participants with pSS during the COVID-19 pandemic. These are outlined in Table 5.

**Table 5:** Outlining additional questions posed to participants to measure the personal impact of COVID-19 pandemic on participant’s daily life.

Question	Answer options
Did you have medical appointments affected during the COVID-19 pandemic?	Yes/no
Please indicated if these were cancelled/delayed/rescheduled	Multiple choice
Please indicate what specialities the appointments related to	Open answer
Over the past year did you experience disruptions to your prescribed medication regime?	Yes/no
Have you noticed a worsening of sicca symptoms due to wearing a mask?	Yes/no

### Statistical analysis

Statistical analysis of data was performed using GraphPad Prism 9.0 for Windows (GraphPad Software, La Jolla, CA, USA). Results from the questionnaires were calculated as per the developer’s instructions. The Wilcoxon matched pairs and Mann Whitney test was used to compare continuous variables. Spearman r was used for correlation analysis. Data is expressed as the mean ± standard deviation. A p value of < 0.05 was considered statistically significant.

### Ethical approval and informed consent

The Research and Ethics Committees of the Royal Victoria Eye and Ear Hospital (RFSS2019) and The Royal College of Surgeons in Ireland (001661) granted ethical approval for this study and the study adhered to the tenets of the Declaration of Helsinki.

## Data Availability

The datasets used and/or analysed during the current study are available from the corresponding author on reasonable request.
